# Secondary Immune Thrombocytopenic Purpura Due to Primary Epstein- Barr Virus Infection

**DOI:** 10.7759/cureus.26112

**Published:** 2022-06-20

**Authors:** Heba Yusuf, Aretha Kou, Claire Zelinskas, Girma Ayele, Johnathan Frunzi, Rediet Tefera Atalay, Miriam B Michael

**Affiliations:** 1 Internal Medicine, HCA Florida Trinity, Trinity, USA; 2 Internal Medicine, Medical Center of Trinity, Trinity, USA; 3 Internal Medicine, Howard University Hospital, Washington DC, USA; 4 Internal Medicine, Howard University, Washingon DC, USA; 5 Internal Medicine, University of Maryland, Baltimore, USA

**Keywords:** infectious mononucleosis, primary ebv infection, severe thrombocytopenia treatment, severe thrombocytopenia, immune thrombocytopenic purpura

## Abstract

A rare complication of infectious mononucleosis is immune thrombocytopenic purpura (ITP). The majority of people affected by Epstein-Barr Virus (EBV) are below the age of 30, while ITP is usually seen with peaks of incidence in the elderly and children. The unique case of an otherwise healthy 22-year-old female will be discussed, with an initial presentation of ecchymosis, rash, and epistaxis, and was subsequently found to have severe thrombocytopenia. The diagnosis of primary EBV infection due to EBV was eventually made, responsive to intravenous (IV) Methylprednisolone. It is important to consider primary EBV infection in the differential diagnosis of a patient who presents with acute thrombocytopenia.

## Introduction

Immune thrombocytopenic purpura (ITP) may present as a primary or secondary form, characterized by an isolated thrombocytopenia - platelet count < 100,000/microliter (mcL) (normal value 150,000-450,000 platelets/mcL) [1]. ITP can present at any age; however, it does have a bimodal distribution with peaks of incidence among children and the elderly [1]. A common side effect of ITP is a bleeding diathesis - associated hematological abnormalities include atypical lymphocytosis [2]. Epstein-Barr virus (EBV) is a common herpes virus affecting at least 90% of the population worldwide before 30 years old [3,4]. Infectious mononucleosis is a syndrome caused by the EBV. Clinical manifestations seen in infectious mononucleosis include fever, pharyngitis, adenopathy, malaise, and atypical lymphocytosis. In some cases, the complications are not accompanied by typical IM symptoms or signs such as cerebellitis, meningitis, optic neuritis, peripheral neuritis, facial nerve palsy, and Gullian-barre syndrome. Encephalitis, splenomegaly and splenic rupture, hepatic failure, myocarditis, or neutropenia-associated sepsis.Impending airway obstruction results from tonsillar lymphoid hyperplasia and edema, and cytopenia. Aplastic anemia and hemophagocytic lymphohistiocytosis are rare life-threatening complications [5].

Severe thrombocytopenia, characterized as platelet count < 20,000/mcL (normal value 150,000-450,000 platelets/mcL), is a very rare complication of primary EBV infection and has been reported incidentally or sporadically [2]. This case report presents a unique case of secondary ITP due to primary EBV infection in an otherwise young, healthy patient who did not present with any viral symptoms before presentation, highlighting the importance of including IM in the differential diagnosis for young patients who present with severe thrombocytopenia [2].

## Case presentation

**Figure 1:**
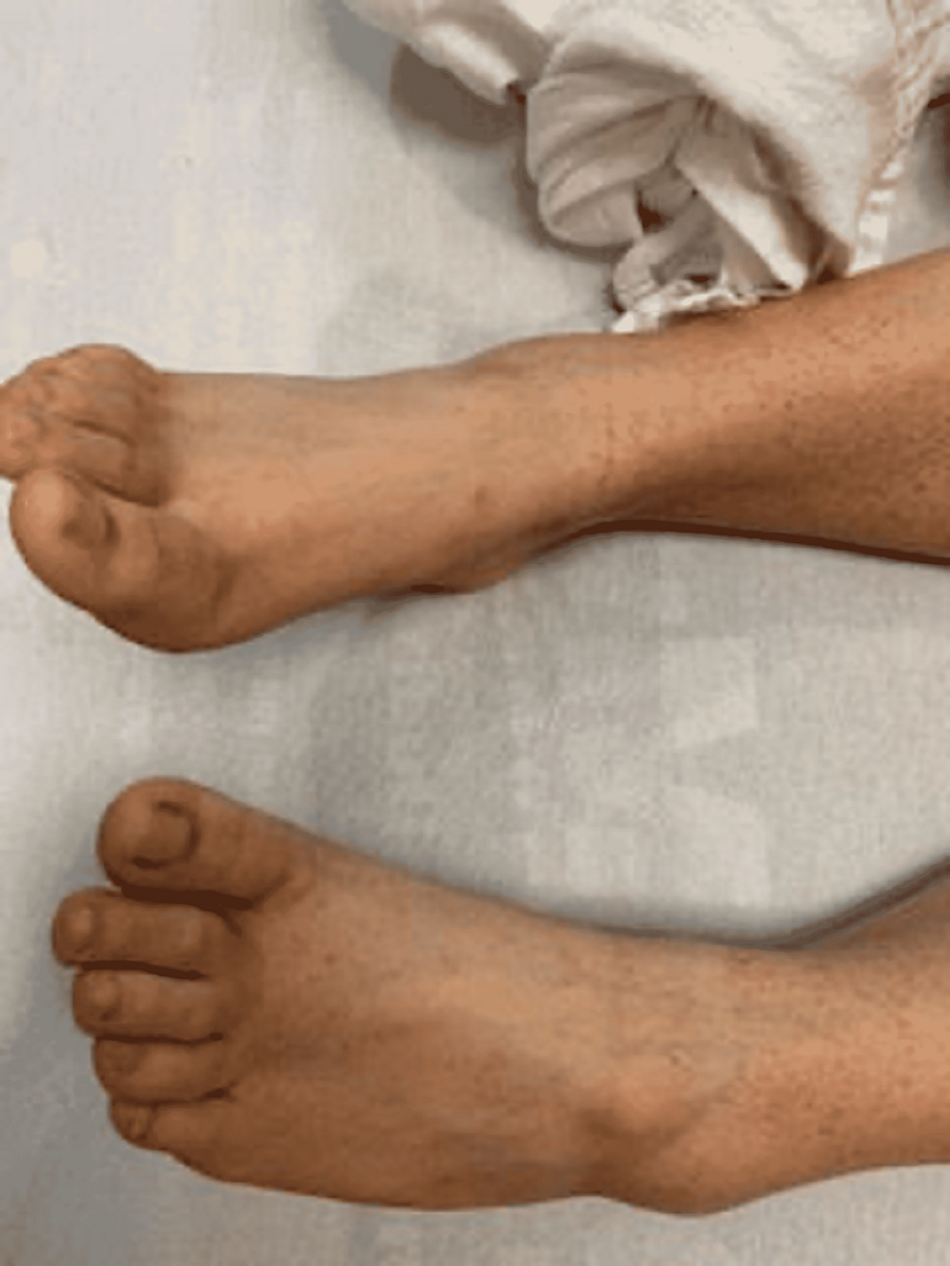
Diffuse petechial rash seen on the patient in the setting of acute EBV infection

**Figure 2:**
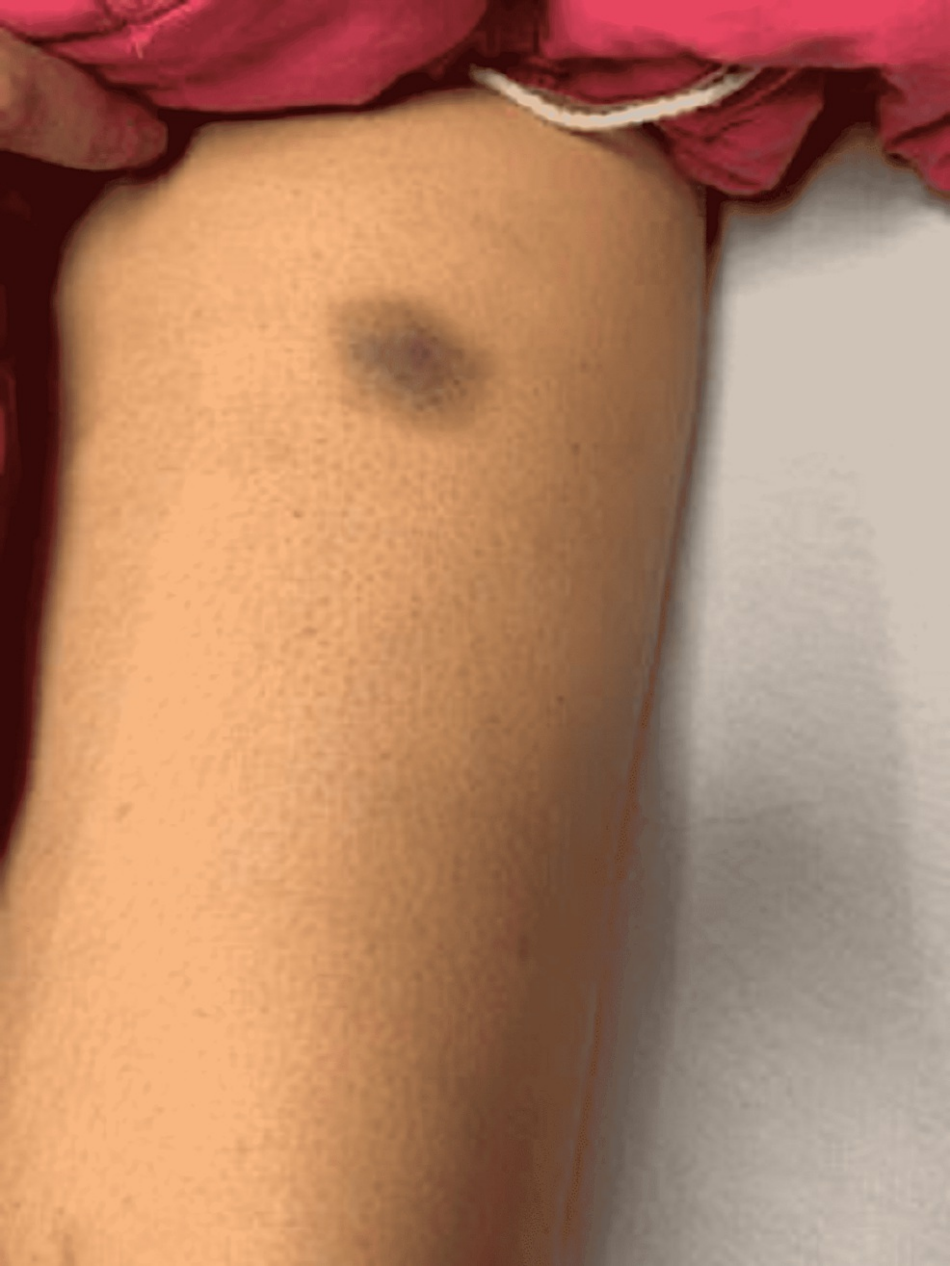
Ecchymosis seen on the patient in the setting of acute EBV infection

**Figure 3:**
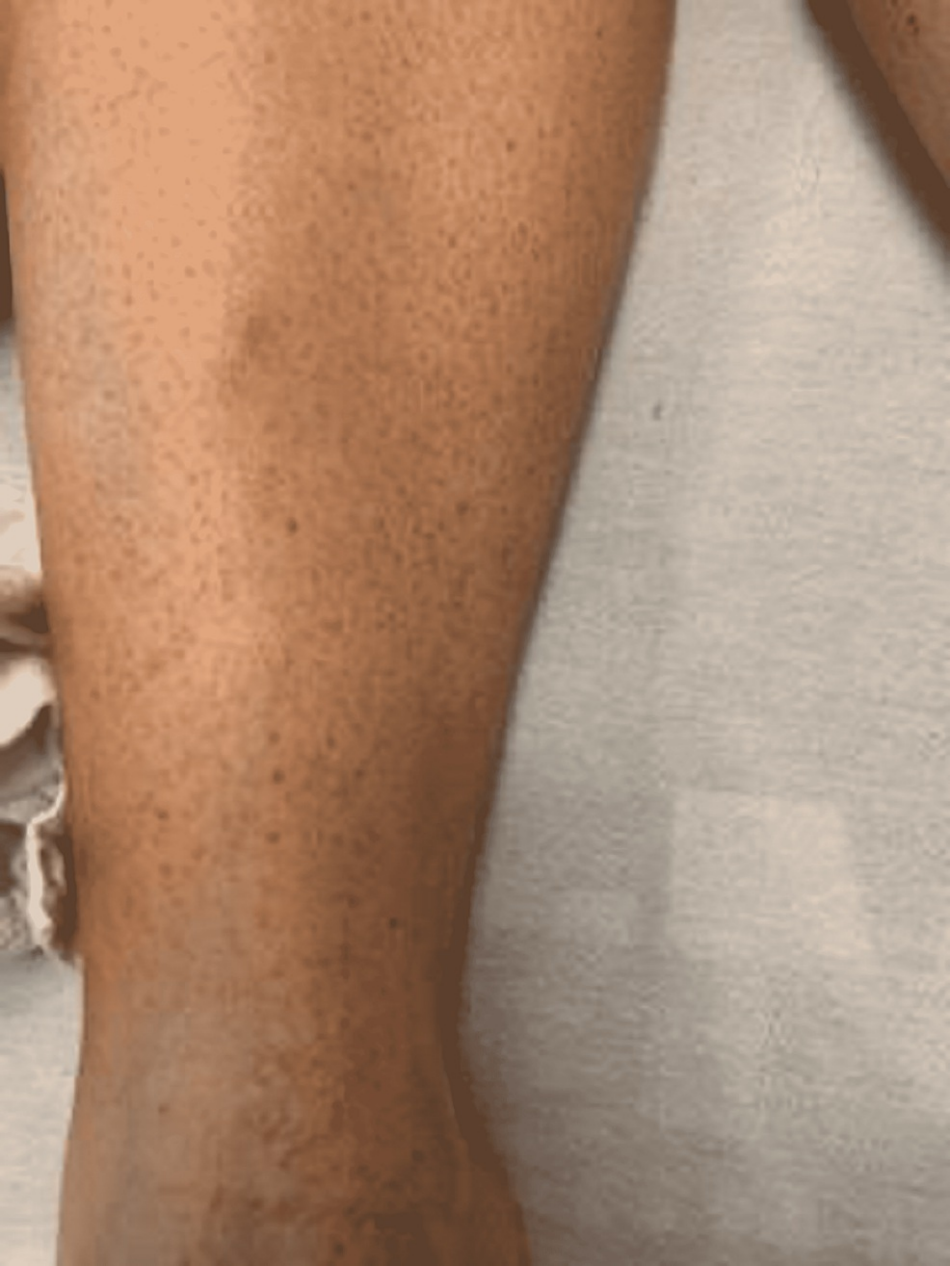
Diffuse petechial rash seen on the patient in the setting of acute EBV infection

A 22-year-old female patient presented with a two-day history of upper extremity bruising, diffuse petechial rash, ecchymosis in bilateral lower extremities, and epistaxis. She was previously healthy and worked as a veterinary technician. The patient took naproxen for three days for headaches the week prior and stopped taking naproxen when she noticed bruising. Initial vital signs were stable. Physical examination was significant for small mucosal hemorrhages on the upper gum/palate, small residual hemorrhage on the lower lip, few ecchymoses, and diffuse petechiae over bilateral lower extremities (Figures 1-3). 

Lab results are summarized in Table 1, which was significant for initial platelet count of 4,000/mcL (normal value 150,000-450,000 platelets/mcL) and white blood cell count of 17380/uL (normal value 4000-10500/uL), lymphocytosis, 13490/uL (normal range 1180-3740/uL), with atypical lymphocytes on peripheral smear suggestive of mononucleosis. Monospot was positive, despite the absence of viral symptoms. EBV serologic titers were positive for both viral capsid antigen immunoglobulin M (IgM) and immunoglobulin G (IgG), consistent with acute mononucleosis. 

**Table 1:** WBC-white blood cell, Plt-platelet, HIV-Human Immunodeficiency Virus, NR-non reactive

Test done	Result	Reference Range
Platelet Count	4,000 plt/mcL, Low	150,000-450,000 plt/mcL
Hemoglobin	14.6 g/dL	11.2-15.7 g/dL
WBC Count	17380/uL, High	4000-10500/uL
Lymphocyte Count	13490/uL, High	1180-3740 /uL
Aspartate aminotransferase	183 unit/L, High	15-37 unit/L
Alanine aminotransferase	288 unit/L, High	30-65 unit/L
Alkaline phosphatase	206 unit/L, High	50-136 unit/L
HIV	NR	
Hepatitis B and C	NR	

She was treated with intravenous (IV) Methylprednisolone 125 mg x1 in the Emergency Department (ED), followed by 60 mg q6 hours with oral pantoprazole and 1 unit platelet transfusion. Laboratory results showed gradual improvement of platelet counts with a peak of 72,000 plt/L and normalization of liver function tests (LFTs) before discharge on day three of hospital stay. Diagnosis of secondary ITP likely due to acute EBV infection/mononucleosis was made. 

## Discussion

ITP is a condition with low platelet numbers due to the destruction of platelets and decreased platelet manufacturing. The proportion of children whose destroyed ITP was associated with documented acute viral infection was 13.3%. ITP is known to be an autoimmune disorder that is identified by its early exploitation of platelets that are triggered by the reticuloendothelial system following sensitization by antiplatelet glycoprotein autoantibodies [6]. Other components of the disorder may include complement-mediated lysis, inadequate thrombopoiesis, or a viral infection that has a detrimental effect on the body's defense mechanisms [6]. Even so, pathogenesis continues to be poorly understood.

The autoimmune system's course of action is assumed to occur within the spleen. In viral disease, the mechanism of thrombocytopenia appears to be multifactorial. The mechanism is usually related to possible deterioration of the immune system that may be caused by antiplatelet antibodies or immune complexes, faulty platelet generation, or an altered reticuloendothelial performance, which are the most common explanations for thrombocytopenia-associated viral diseases [6]. Nevertheless, no analysis has revealed EBV protein expression and CMV late protein expression in the spleen [7].

Dysfunctional cellular immunity is considered to be essential to the pathophysiology of Epstein-Barr virus-related ITP. The pathophysiology of thrombocytopenia due to EBV is multiple. The first hypothesis is the formation of autoantibodies against platelet glycoproteins IIb-IIIa and Ib-IX, but autoantibodies are only seen in 40% of patients [4]. Another theory is hypersplenism-induced platelet sequestration, however, normal platelet count has been seen in cases with splenomegaly, and like this case, many patients with serious thrombocytopenia can have a normal-sized spleen [4]. Vascular damage, hemophagocytic lymphohistiocytosis, and EBV presence in the bone marrow are some of the different theories for EBV-induced thrombocytopenia [4].

This young, otherwise healthy patient presented with petechiae, ecchymosis, and epistaxis that responded well to corticosteroid treatment. She had no viral symptoms suggestive of acute mononucleosis. She presented to the ED due to bleeding diathesis likely due to severe thrombocytopenia with an initial platelet count of 4,000 plt/L. Bleeding was likely exacerbated due to the recent use of naproxen, which would have altered platelet function [3]. Still, severe thrombocytopenia from secondary ITP due to primary EBV infection is primarily the cause of acute bleeding diathesis (petechiae, ecchymosis, etc.). A study out of Japan showed that ITP associated with EBV had a longer course and resolved more slowly than those without EBV infection usually a difference of 10 days even with treatment causing increased morbidity [8].

First-line treatment for acute ITP as per the recommendation of the National Institute for Health and Care Excellence (NICE) included steroids and/or Intravenous immunoglobulin (IVIG). For life-threatening bleeding, platelet transfusion is used as a temporary measure. IVIG infusion preceding platelet transfusion may increase platelet survival through the immune-modulatory mechanism as per some studies. Additionally, some studies have shown that the thrombocytopenia that is caused by EBV may be refractory to steroid treatment and high-dose IVIG has shown a rapid increase in the platelet count [9]. Even though IVIG is effective it comes with adverse effects like immediate flu-like symptoms, these side effects can be prevented by decreasing the infusion rate or pre-infusion treatment with non-steroidal anti-inflammatory drugs (NSAIDs) [4]. 

The goals of ITP therapy are to prevent severe bleeding episodes and maintain a target platelet count of at least 20,000-30,000 plt/L. For the case of EBV infection complicated by severe thrombocytopenia, corticosteroids have been used even though several weeks are needed to increase the platelet count to 30,000 plt/L [6]. Though this patient showed rapid platelet recovery with corticosteroid treatment and platelet transfusion, various platelet recovery times have been documented at times lasting up to six weeks [3,4]. For cases that are refractory to steroids, IVIG was effective [5]. Still, there is a lack of critical evaluation of treatment effectiveness [3,4].

## Conclusions

Secondary ITP is a rare complication of acute primary EBV infection, which can present with a bleeding diathesis in the setting of severe thrombocytopenia despite the absence of viral symptoms at any age. As there are different theories thought to explain the pathophysiology of ITP, many triggers have been identified to precipitate this autoimmune reaction. This case highlights the significance of acute primary EBV infection in a young, healthy patient with severe thrombocytopenia. Although rare, secondary ITP due to acute primary EBV infection should be evaluated for, especially in EBV prevalent populations. Despite varied treatment response times and efficacy, in this case, the young patient responded rapidly to mainstay therapy with corticosteroids, which avoided serious bleeding complications. Hence, it is essential to consider EBV infection in the differential diagnosis of a patient who presents with acute thrombocytopenia. 
